# Tolerance to High Temperature Extremes in an Invasive Lace Bug, *Corythucha ciliata* (Hemiptera: Tingidae), in Subtropical China

**DOI:** 10.1371/journal.pone.0054372

**Published:** 2013-01-24

**Authors:** Rui-Ting Ju, Lei Gao, Xu-Hui Zhou, Bo Li

**Affiliations:** 1 Department of Plant Protection, Shanghai Institute of Landscape Gardening Science, Shanghai, China; 2 Coastal Ecosystems Research Station of the Yangtze River Estuary, Ministry of Education Key Laboratory for Biodiversity Science and Ecological Engineering, Institute of Biodiversity Science, Fudan University, Shanghai, China; La Trobe University, Australia

## Abstract

Biological invasions are predicted to be more frequent as climate change is increasing its positive impact on the prevalence of invasive exotic species. Success of insect invaders in different temperature zones is closely related to their tolerance to temperature extremes. In this study, we used an exotic lace bug (*Corythucha ciliata*) as the study organism to address the hypotheses that an insect species invading a subtropical zone from temperate regions has a high capacity to survive and adapt to high temperatures, and that its thermal tolerance plays an important role in determining its seasonal abundance and geographic distribution. To test these hypotheses, the effects of heat shock on the survival and reproduction of *C. ciliata* adults were assessed in the laboratory. Adults were exposed to 26 (control), 35, 37, 39, 41, 43, and 45°C for 2 h, and then were transferred to 26°C. Heat-shock temperatures ranging from 35 to 41°C did not significantly affect survival pattern, longevity, and fecundity of adults, but heat shock at 43 and 45°C significantly reduced these traits. Exposing parent females to heat-shock treatments from 35 to 41°C did not significantly affect the hatching rate of their eggs, survival of the nymphs, and the proportion of female *F*_1_ progeny, while no progeny were produced with treatments of 43 and 45°C. The results indicate that *C. ciliata* can tolerate high temperatures less than 41°C, which may contribute to its expansion into the lower latitudes in China where its hosts (*Platanus* trees) are widely planted. Our findings have important implications for predicting seasonal abundance and understanding invasion mechanisms of this important urban invader under climate change.

## Introduction

Climate change has become one of the greatest threats to global biodiversity and ecosystem functioning [1]. Global surface temperature has increased by 0.75°C since 1850 and is projected to increase another 1.1 to 6.4°C by the end of the 21st century as a consequence of anthropogenic buildup of greenhouse gases in the atmosphere [2]. Global warming is believed to cause more frequent and extreme heat waves in many parts of the world. Over the past 60 years, extremely high temperature events around the world have increased by 40% [3], and heat waves are predicted to increase their duration, frequency, and/or intensity with a 90–99% probability [2]. The extreme hot temperature largely affects individual species, biological communities, and ecosystems. Under higher temperature, species may either show the production of an alternative phenotype from a given genotype, or may show some other forms of reaction to the environmental conditions. The response referred to phenotypic plasticity, which has been widely discussed for many organisms including insects [4], [5], [6], [7], [8], [9].

Insects are ectotherms, and temperature therefore greatly affects their behavior, distribution, development, survival, and reproduction [10], [11], [12]. Insects maintain their physiological functions only within a specific range of temperature, and temperature extremes may reduce their survival and reproduction [13]. Temperature can limit geographic range of herbivorous insects by causing direct mortality or by limiting the distribution of host plants [14], [15]. The increasingly abrupt heat waves caused by climate change might increase the frequency of insects' exposure to high temperatures that may alter physiological tolerance to environmental stress and may, therefore, cause the changes in biology and distribution of insects [16]. This effect can be further enhanced by other abrupt climate events (e.g., drought and storm) under climate change [9], [17], [18].

The ability of an exotic insect species to overcome stressful temperatures is crucial to its establishment and spread in new regions [19]. Given that climate warming may facilitate biological invasions [20], increasing attention has been paid to the effects of high temperatures on invasive exotic insects [16], [21], [22]. It is believed that invasive insects with high thermal tolerance are likely to be favored by climate warming [23], [24], [25]. If an invasive insect can tolerate high temperature extremes, climate warming is likely to accelerate its spread [25]. Therefore, it is important to understand thermal tolerances of new invasive insects and how the traits affect their further invasions.

The sycamore lace bug, *Corythucha ciliata* (Say) (Hemiptera: Tingidae), feeds on *Platanus* trees and is a recently recognized invasive pest in China [26]. Native to temperate regions of North America, this species has invaded the temperate and subtropical zones, including Europe, Australia, South America, and East Asia [27]. In China, *C. ciliata* was first found in Hunan Province in 2002 and then spread to 11 other provinces between latitudes 26°N and 37°N over the last decade [28]. *C. ciliata* has caused the most damage in subtropical China, particularly in the Yangtze basin, where it has infested almost 70% of host trees [29], [30]. *C. ciliata* is an oligophagous insect with a short life cycle, and its hosts (*Platanus* spp.) are the main trees planted along the streets in China. Because *Platanus* trees are widely planted in China, the spread of this pest will not be limited by host availability but might depend on its ability to adapt to environmental conditions, especially to temperature. Despite previous work on cold tolerance of *C. ciliata* [28], [30] and other effects of temperature on this species [12], [29], there is generally a lack of quantitative data on how its reproduction and fitness are affected by high temperatures.

**Figure 1:**
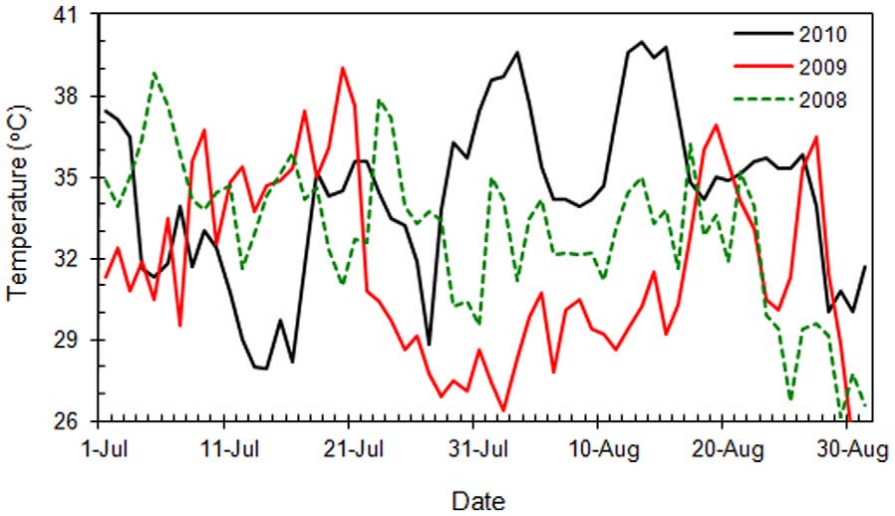
Daily maximum temperatures during summer in Shanghai, China in 2008, 2009, and 2010. Data were obtained from the official website of Shanghai Meteorological Bureau at www.smb.gov.cn. The weather station providing these data is located in Longhua Town, Xuhui District, Shanghai (31.17°N, 121.45°E).

A previous study demonstrated that *C. ciliata* could not develop from egg to adult when cultured at constant temperatures above 36°C [29]. Under natural conditions, however, *C. ciliata* is occasionally subjected to higher temperatures (>36°C), especially during the summer [31]. In Shanghai (serious damage by *C. ciliata* has occurred in these years), for example, daily high temperatures in July and August can exceed 36°C for many consecutive days, and maximum temperatures have been as high as 40°C over the recent years (Figure 1) (temperatures in the canopies of *Platanus* trees are usually 0.5–1.0°C lower than the air temperatures recorded by meteorological stations). Furthermore, researchers predict that, in subtropical China where *Platanus* trees are widely distributed, summer temperatures may reach or exceed 40°C with increasing frequency in the next 50–75 years [32]. In these regions, the abundance of *C. ciliata* is higher in summer than in spring and late autumn. Another previous study of ours showed that, when compared with that at 26°C (the optimal temperature for survival), survival of eggs, nymphs, and adults of *C. ciliata* was not affected by a short exposure at temperatures ≤41°C [12]. We hypothesize that this species has a great capacity to adapt to high temperatures, and that high temperature tolerance plays an important role in determining its seasonal abundance and geographic distribution.

Here, we investigated whether tolerance to high temperatures might contribute to the invasiveness of *C. ciliata*. We conducted a laboratory study to assess the effects of short-term high temperatures on *C. ciliata*. We answered the following questions: (1) Is the survival pattern of *C. ciliata* adults affected by short-term heat stress (heat shock)? (2) How is the reproductive capacity of *C. ciliata* adults affected by heat shock? (3) Is the fitness of *C. ciliata* progeny influenced by heat shock experienced by the parent females?

## Materials and Methods

### Insect culture

A laboratory colony of *C. ciliata* was established in May 2010 from adults collected from London plane trees (*Platanus*×*acerifolia*) in Shanghai, China (31.2°N, 121.5°E). The insects were reared on leaves of *P.*×*acerifolia* in a mesh cage (50×30×30 cm). One branch with 5–6 leaves was placed in the cage. The cut end of the branch was in a water-filled bottle, and the branches were changed as needed to ensure a steady food supply. The stock culture was maintained in the laboratory at 26±0.5°C with a relative humidity (RH) of 80±5% and a 14 h∶10 h (L∶D) photoperiod. Newly emerged adults of *C. ciliata* (<24 h old) of the *F*_1_–*F*_2_ generation were used in the experiments.

### Adult survival pattern immediately after short-term heat stress (experiment 1)

Newly emerged adults of *C. ciliata* were placed individually in a closed Petri dish (diameter: 9 cm) and then were heat shocked at 35, 37, 39, 41, 43, and 45±0.5°C for 2 h in climatic incubators (MIR 350H, Sanyo Electric Co. Ltd., Osaka, Japan). The temperatures were selected based on possible high temperature extremes in the Yangtze basin of China, where populations of *C. ciliata* are established. The 2-h heat-shock time was selected based on the longest duration of the temperature extremes in this region. At the end of the exposure, adults were transferred to 26°C and a 14 h∶10 h (L∶D) photoperiod. The adults were allowed to recover for 2 h, and then the numbers of adults alive and dead were determined. According to a pilot test, this was sufficient time for recovery for those that would eventually recover; recovery did not increase with recovery times >2 h. The adults were considered dead if no appendage moved after the appendages were touched with a brush. Each treatment was replicated three times (with at least 30 individuals for each replicate). For the control, adults were maintained at 26°C throughout the experiment.

### Adult longevity and reproduction after short-term heat stress (experiment 2)

Newly emerged adults were subjected to heat shock and a recovery period as described for experiment 1. The surviving adults from each treatment were paired (one male and one female), and each pair was placed on one leaf of *P.*×*acerifolia* in a closed Petri dish (diameter: 9 cm) with a wet filter paper. The leaf acted as a food source and oviposition site. After oviposition, the leaf with attached eggs was removed, and each pair of adults was then supplied with a new leaf. Leaves with eggs were removed and replaced daily until the adults died. The eggs on the leaves were counted with a binocular stereoscope (MZ 16A, Leica Microsystems Ltd., Wetzlar, Germany). As described in our previous study [29], the following data were collected: length of the preoviposition period, length of the oviposition period, adult longevity, and adult fecundity. At least 30 pairs of adults were assayed per treatment. Untreated adults maintained at 26°C served as the control.

### Effects of short-term heat stress applied to parent females on their *F*_1_ progeny (experiment 3)

Newly emerged males and females were subjected to heat shock and placed on leaves in Petri dishes as described for experiment 2. The eggs produced (*F*_1_ progeny after heat shock) together with the leaves were maintained in a Petri dish (diameter: 9 cm) at 26°C, 80±5% RH, and a 14 h∶10 h (L∶D) photoperiod. The eggs were examined 10 days after oviposition, and the hatched nymphs were counted. The eggs were continuously examined every 2 days until no nymphs hatched after 5 successive days. At the end of each census interval, the leaves with nymphs were placed in climatic chambers until completion of adult eclosion (when a nymph appeared on the leaf, it was transferred to a new Petri dish with the same food source and environmental conditions described in the earlier experiment). After adult emergence, the adults were immediately sexed. Survival from nymph to adult and the percentage of females were calculated. At least three replicates with more than 30 individuals per replicate were evaluated for each treatment. Eggs produced by adults maintained at 26°C were used as the control.

### Data analysis

Survival pattern was accessed by the curves that describe the age-specific survivorship (*l_x_*) with days of the adults after exposed heat shock [33]. The effects of heat-shock treatments on longevity, reproductive parameters in parent adults, and fitness parameters in *F*_1_ progeny were tested by one-way analysis of variance (ANOVA), and means were compared using Tukey's test (*P*<0.05). Statistical analyses were performed with SPSS 15.0 [34]. All percentages were log-transformed prior to statistical analyses.

Demographic parameters were estimated from the survival and fecundity data of adults after exposure to heat-shock treatments as described [35]. The following parameters were calculated:

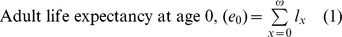

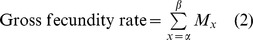

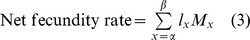

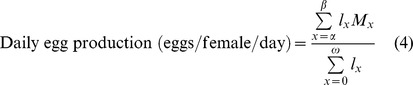

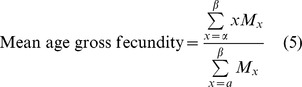

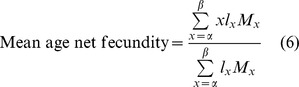

where *x* is the age in days; *α* is the age at the start of reproduction; *β* is the age at the end of reproduction; *ω* is the last possible day of life; *l_x_* is the proportion of individuals surviving to age *x*; *M_x_* is the average number of eggs laid by (living) females of age *x*; and *e_0_* is the average number of adult-days lived by the average adult from age 0 to death.

## Results

### Survival pattern of adults after short-term heat stress (experiment 1)

**Figure 2:**
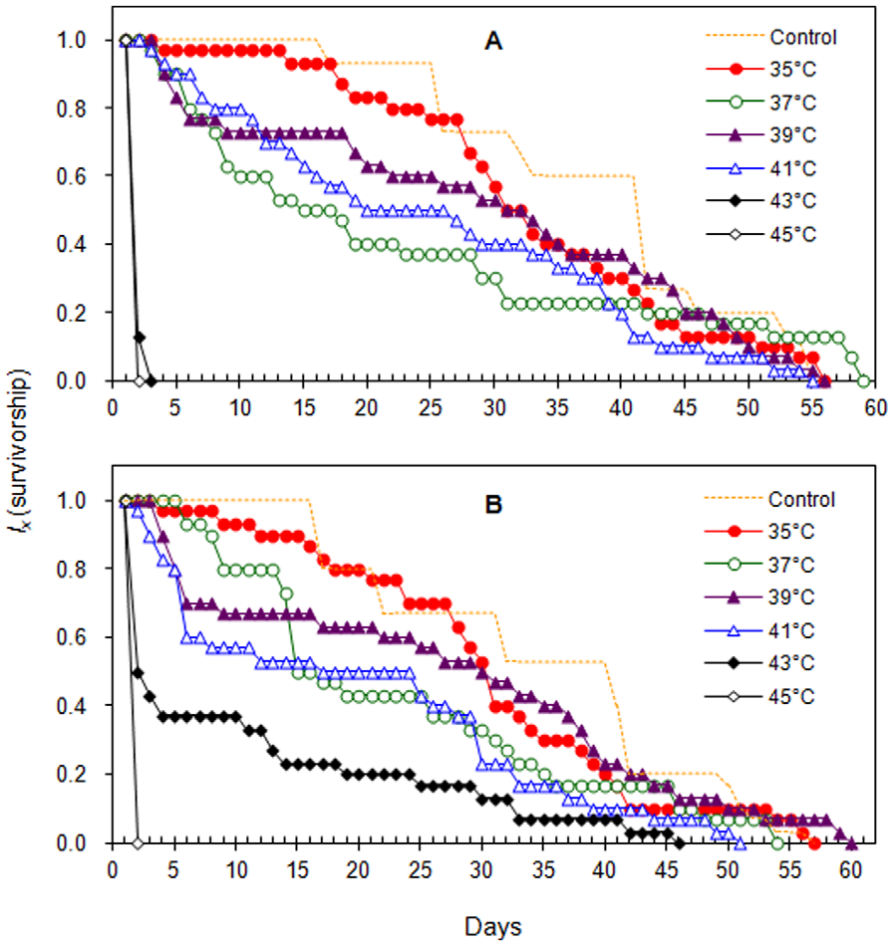
Age-specific survivorship (*l_x_*) curves of male and female *C. ciliata* adults after exposure to heat-shock treatments (2 h) in experiment 2; adults that were not exposed to heat shock but were kept at 26°C served as the control. (A) ♂♂, (B) ♀♀.

Heat-shock remarkably affected age-specific survivorship (Figure 2). Adults exposed to mild heat-shock temperatures (35–41°C) exhibited a type II age-specific survivorship curve (in which the drop in survival was interrupted by plateaus) while those exposed to the higher heat-shock temperatures (43 and 45°C) initially exhibited a type II curve but progressively exhibited a type III curve (in which survival dropped without substantial plateaus). The pattern of the age-specific survivorship curve did not differ between the sexes for most heat-shock treatments (Figure 2). In the control, adults did not begin to die until they were 17 days old ( = adult day 17). At the lower heat-shock temperatures (35–41°C), adults began to die when they were 2 to 5 days old. At heat-shock temperatures of 43 and 45°C, adults began to die immediately, and all adults died when they were 3 days old. The longest lived individual male died when 59 days old and had been exposed to 37°C, while the longest lived individual female died when they were 60 days old and had been exposed to 39°C. The longest lived untreated male and female died when they were 55 and 57 days old, respectively. In all heat-shock treatments, the survival rate of adults of both sexes was lower than 20% once they were older than 44 days.

### Longevity and reproduction after short-term heat stress (experiment 2)

Adult longevity (for both males and females) differed among heat-shock treatments (Table 1). When the heat-shock temperatures were lower than 43°C, the longevity ranged from 30 to 40 days for all adults and was not significantly different from that of the control (34.73 days for males and 33.20 days for females at 26°C). Longevity of males and females declined to 1–3 days after heat-shock at 43°C. In most treatments, the average lifespan was longer for males than females but this difference was not statistically significant among the temperature treatments (Table 1) (*t*-test, *P*>0.05).

**Table 1:** Reproductive parameters and longevity of *C. ciliata* adults after exposure to heat-shock treatments.

Temperature (°C)	Longevity (d)^a^		Preoviposition period (d)	Oviposition period (d)	Fecundity (eggs/♀)
	♂♂	♀♀			
26^b^	34.73±2.71 a^c^	33.20±4.49 a	5.53±0.46 c	11.60±0.35 c	273.00±9.35 a
35	34.58±1.48 a	33.16±1.48 a	12.04±0.77 a	14.96±1.41 bc	266.44±5.69 a
37	36.38±2.18 a	34.00±2.05 a	10.89±1.06 ab	18.07±2.62 ab	278.08±9.92 a
39	37.52±2.44 a	35.90±2.61 a	8.56±0.82 b	21.94±1.53 a	263.62±8.98 a
41	33.56±2.54 a	30.78±2.74 a	9.93±0.95 ab	14.14±1.99 bc	270.85±7.87 a
43	1.13±0.06 b	2.77±0.62 b	–	–	–
45	–	–	–	–	–
*F*	58.21	39.18	8.43	5.40	0.45
*d.f.*	5, 163	5, 164	4, 90	4, 82	4, 82
*P*	<0.0001	<0.0001	<0.0001	0.0007	0.7696

a Means between sexes were not significantly different (*t*-test: *P*<0.05).

b Adults in this treatment were not exposed to heat shock but were kept at 26°C (control).

c Values are means ± SE. Means within a column followed by different letters are significantly different (Tukey's test: *P*<0.05).

For most heat-shock treatments, adult life expectancy (*e_0_*) was higher for males than for females (Table 2). Adult life expectancy ranged from 32.32 (35°C) to 1.13 days (43°C) for males and from 29.82 (35°C) to 9.79 (43°C) for females. Adult life expectancy for the untreated adults was estimated to be 37.56 days for males and 33.97 days for females.

**Table 2:** Demographic parameters^a^ of *C. ciliata* adults after exposure to heat-shock treatments.

Temperature (°C)	Adult life expectancy (days)		Gross fecundity rate	Net fecundity rate	Daily egg production	Mean age gross fecundity	Mean age net fecundity
	♂♂	♀♀					
26^b^	37.56	33.97	270.34	189.71	6.73	23.14	19.39
35	32.32	29.82	263.98	195.41	6.75	20.97	19.36
37	22.18	22.76	230.77	123.30	6.81	19.22	16.14
39	28.06	26.38	233.48	140.75	5.75	19.39	18.09
41	24.20	19.14	232.08	115.95	5.42	16.60	15.21
43	1.13	9.79	–	–	–	–	–
45	–	–	–	–	–	–	–

a Demographic parameters were estimated as described in Carey (1993) [35].

b Adults in this treatment were not exposed to heat shock but were kept at 26°C (control).

The average preoviposition period was significantly longer (3–7 days) for females exposed to heat-shock treatments than for females kept at 26°C (5.53 d for untreated control) (Table 1). The oviposition period did not differ between the control (11.60 days) and heat shock at 35°C (14.96 days) and 41°C (14.14 days), but that was greater (1.55 times longer) with heat shock at 37°C and 39°C than for the control (Table 1). The mean number of eggs laid per female did not differ among heat-shock treatments (35–41°C) and the control (273.00 eggs per female) (Table 1).

The demographic parameters of female oviposition and fecundity after heat shock are shown in Table 2. After heat-shock treatments, the gross fecundity rate (230.77–263.98) was slightly lower than that of the control (270.34) but no consistent patterns were observed for net fecundity rate (115.95–195.41) or daily egg production (5.42–6.75). The daily egg production was similar to that of the control at 35 and 37°C, but it began to decline when the heat-shock temperatures increased to 39°C or higher. Overall, the mean age gross fecundity of female individuals that survived until the theoretical last day of their life expectancy decreased with the increasing heat-shock temperature, and was estimated to be 16–24 days. If the mortality of females was taken into consideration (mean age net fecundity), this value declined by 1–4 days (Table 2).

**Figure 3:**
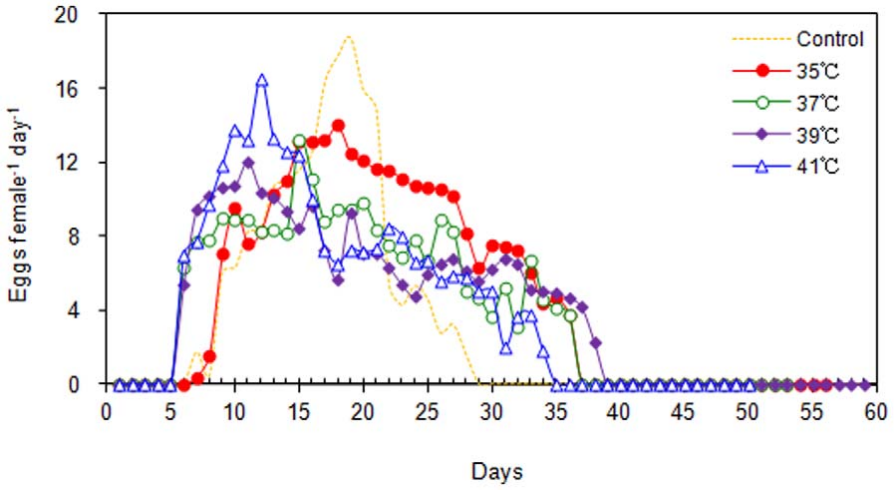
Number of eggs per female per day (*Mx*) after *C. ciliata* females were exposed to heat-shock treatments (2 h) in experiment 2; females that were not exposed to heat shock but were kept at 26°C served as the control.

At all heat-shock temperatures, oviposition started when adults were 6 to 7 days old, and the number of eggs laid per day progressively increased for the next 7 days or so (Figure 3). The oviposition curve (*M_x_*) differed for heat-shocked and untreated female adults. The number of eggs deposited per day by untreated females peaked at adult day 19, 13 days after the beginning of oviposition, and then progressively declined. In contrast, the increase in heat-shock temperature resulted in a displacement of the oviposition curve to the left (eggs peaked at adult day 18, 15, 11, and 12 for heat-shock temperatures of 35, 37, 39, and 41°C, respectively). After the peak, the number of eggs laid by heat-shocked females exhibited increasing day-to-day variability.

### Effects of short-term heat stress applied to parent females on their *F*_1_ progeny (experiment 3)

**Figure 4:**
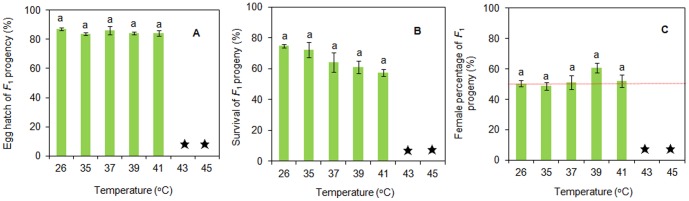
Biological parameters of *C. ciliata F*_1_ progeny whose parent females had been heat shocked in experiment 3; parents that were not exposed to heat shock but were kept at 26°C served as the control. Values are means ± SE. ★ indicates no *F*_1_ progeny. Values with the same letters are not significantly different (Tukey's test: *P*<0.05). (A) Egg hatch, (B) Survival from nymphs to adults, (C) Female percentage.

No *F*_1_ progeny were reproduced after parent adults were heat shocked at 43 and 45°C. The egg hatching rate of *F*_1_ progeny of *C. ciliata* did not significantly differ among the treatments after heat shock at 35–41°C (*F*_4, 57_ = 0.64, *P* = 0.571), ranging from 84.0% to 86.2% and approximating that of the control (86.9%) (Figure 4–A). The survival rate of the progeny of *C. ciliata* (the percentage of nymphs that developed into adults) was not significantly affected by the heat-shock treatments at 35–41°C (*F*_4, 53_ = 2.36, *P* = 0.068). But compared with the control (74.8%), the survival rates of the progeny slightly declined to 57.3% when the temperature increased to 41°C (Figure 4–B). Of the nymph progeny that developed into adults, the percentage of females did not differ among the heat-shock treatments at 35–41°C (*F*_4, 64_ = 1.35; *P* = 0.262). About 50% of the progeny developed into females in the heat-shock treatments and the control (Figure 4–C).

## Discussion

Generally, a brief exposure to a high temperature injures insects, and the lethal temperature is usually between 40 and 50°C depending on the species and their life stages [36]. Our previous results showed that survival of both males and females of *C. ciliata* did not decline significantly until the heat-shock temperature increased to 43°C or higher, and the survival responses to the heat shock were generally similar for both sexes (female and male), suggesting that *C. ciliata* adults have a robust tolerance to temperatures up to 41°C [12]. In another study with invasive insects in the family Hemiptera, survival of adults was not significantly affected by the high temperatures below 41°C for *Bemisia tabaci*, and by the high temperatures below 37°C for *Trialeurodes vaporariorum*. The females of both species, however, were more tolerant to high temperatures than males [22]. These reports clearly demonstrate that heat tolerance differs among insect invaders even at the same family.

The heat-shock temperatures significantly affected the shape of the age-specific survivorship curve of both sexes of *C. ciliata*. At lower heat-shock temperatures (35–41°C), survival tended to decline in steps (with 2- to 10-day plateaus during which survival did not decline, i.e., a type II survivorship curve). Higher heat-shock temperatures (43 and 45°C) tended to change this relationship to the one with no plateaus (i.e., a type III survivorship curve). A possible explanation for the plateaus is that the insect is capable of surviving a series of non-lethal lesions but after some time the lesions accumulate to a critical level and cause death [13]. The absence of plateaus at the higher temperatures might indicate that lethal lesions develop more rapidly at higher temperatures or that any healing of lesions that may occur at lower temperatures does not occur at higher temperatures [10], [13].

In addition to the effects on insect survival, thermal stress can affect the reproduction of insects. Our previous study showed that *C. ciliata* adults did not lay eggs at constant high temperatures above 36°C [29]. In this study, fecundity was similar for *C. ciliata* adults that were not subjected to heat shock from 35 to 41°C, suggesting that this species can tolerate a short duration of exposure to high temperatures ≤41°C. *C. ciliata* ceased depositing eggs after heat shock at 43°C because of heat injury and after heat shock at 45°C because of 100% mortality. Similar results were also found in some other incomplete metamorphosis insect species. For example, heat shock drastically reduced reproductive output of *Aphidius avenae* females [37] and *T. vaporariorum* females [22]. Research on the collembolan *Orchesella cincta* indicated that heat shock also affected male reproductive success in an epimorphosis Hexapoda species [38]. The effect of heat stress on fecundity in insects may be due to the injury of oocytes, interference with ovarian development, reduction in male fertility because of direct injury to the testes and sperm, or a decrease in sexual attractiveness between males and females [39], [40], [41], [42], [43]. In our fecundity and reproductive experiments, because both males and females were subjected to heat-shock temperatures, we could not determine whether the reduction in fecundity and the changes in reproductive period resulted from the damage to both sexes or to only one sex.

Previous studies have shown that if an insect survives exposure to heat stress, fitness is often reduced by the changes in survival, development, or reproduction at later stages and even in the offspring [42], [43], [44], [45]. In our study, heat shock (≤41°C) did not affect oviposition by *C. ciliata* or the hatching rates of their *F*_1_ progeny. Although heat shock slightly reduced the survival of *F*_1_ progeny, the difference was not significant among the treatments; survival rate was nearly 60% when the parent females were subjected to heat-shock temperatures ≤41°C. Previous studies suggest that changes in sex ratios may affect the insect population dynamics by interfering in mating or by reducing female emergence [46], [47]. Our experiment showed that the *F*_1_ sex ratio of *C. ciliata* was not significantly affected after the parent females were heat shocked at ≤41°C. This information indicates that high summer temperatures (≤41°C) might not reduce the population size of *C. ciliata*. Nevertheless, research is needed to determine whether and how the *C. ciliata* life-history and phonological parameters are altered under hot conditions in the field.

When invading new areas, invasive insects inevitably face environmental conditions that differ from those in their native range. Environmental adaptability, especially a strong resistance to high and cold temperatures, is a common feature of successful invaders [19], [22]. Many organisms live in variable thermal environments, which pose substantial challenges to their survival and reproduction. Therefore, the ability of an insect to overcome thermal stress, together with other factors, plays an important role in determining distribution of the species [48]. As a species native to temperate regions, *C. ciliata* is extremely cold tolerant; it withstands temperatures as low as −23°C in the USA [49], and adults in China have a supercooling point of −17°C [50]. Our results show that *C. ciliata* can also tolerate high temperatures ranging from 35 to 41°C if the duration of exposure is not longer than 2 h. Host plants of *C. ciliata*, *Platanus* trees, are widely planted in both temperate and subtropical regions of China. In subtropical China, high temperature extremes commonly exceed 40°C in the summer; temperatures greater than 40°C have been recorded in Chongqing, Wuhan, and Nanjing, and higher temperature extremes may occur in next half century [32]. Within the canopies of the host trees in these regions, however, the highest daily temperature has seldom exceeded 41°C over recent years, and the high temperature extremes have usually lasted for not longer than 2 h during the summer [12]. Obviously, the current high temperature extremes have failed to prevent *C. ciliata* from spreading in subtropical China. The data on both cold and heat tolerance demonstrate that *C. ciliata* has a wide ecological amplitude in relation to temperature. Although the distribution of *C. ciliata* in China now covers only the region from 26° to 37° latitude [28], its temperature tolerance in combination with the wide planting of its host (*Platanus* trees) suggest that this pest may continue to spread unless an effective management strategy is implemented.

The current climatic trend is toward increasing temperatures and increasing variability, with extreme climatic events (heat waves, cold snaps, droughts, floods) becoming more frequent [51]. Growing recognition of the importance of climate change has triggered a number of recent studies focused on the impacts of extreme climatic events on the sensitivity, adaptation, and evolution of organisms [52], [53], [54]. Climate warming has strongly affected all life-history and phonological parameters of insect herbivores [47], [48], [55]. Detailed studies from several insect species have shown that these species tend to complete more generations per year and spread to higher latitudes as global warming continues [56], [57], [58], [59], [60]. If climatic anomalies become more frequent, the insects may suffer from deleterious effects of high temperatures in the late summer (the hottest season): they may experience increased mortality, difficulty in moulting, reduced proportion of females, reduced fecundity, and a reduced life span [47]. Only those insect species that have robust tolerances to high temperatures can adapt to the heat waves under climate warming.

There are few studies regarding the tolerance to high temperature extremes of insect species that are native to temperate zones and are spreading to tropic or subtropic zones. In the current paper, we provide data for this kind of invasive species. *C. ciliata* is an important invasive pest that is spreading from relatively cold regions to relatively hot regions and that is now distributed throughout Eastern and Middle China. By measuring the effects of high temperature extremes on survival, reproduction, and fitness, we illustrate that although *C. ciliata* is native to temperate areas, it can tolerate the high temperatures in subtropical regions. We suggest that great heat tolerance has enabled its invasion of subtropical areas.
